# Yoga effectively reduces fatigue and symptoms of depression in patients with different types of cancer

**DOI:** 10.1007/s00520-020-05794-2

**Published:** 2020-10-07

**Authors:** Teresa Zetzl, Agnes Renner, Andre Pittig, Elisabeth Jentschke, Carmen Roch, Birgitt van Oorschot

**Affiliations:** 1 Interdisciplinary Center, Palliative Medicine, University Hospital Würzburg, Josef-Schneider-Str. 11, B1, 97080 Würzburg, Germany; 2 Department of Psychology (Biological Psychology, Clinical Psychology, and Psychotherapy), University of Würzburg, Würzburg, Germany; 3 Center of Mental Health, University of Würzburg, Würzburg, Germany

**Keywords:** Yoga, Complementary alternative medicine, Mind-body intervention, Fatigue, Depression, Quality of life

## Abstract

**Purpose:**

Examine the effects of an 8-week yoga therapy on fatigue in patients with different types of cancer.

**Methods:**

A total of 173 cancer patients suffering from mild to severe fatigue were randomly allocated to yoga intervention (*n* = 84) (IG) versus waitlist control group (CG) (*n* = 88). Yoga therapy consisted of eight weekly sessions with 60 min each. The primary outcome was self-reported fatigue symptoms. Secondary outcomes were symptoms of depression and quality of life (QoL). Data were assessed using questionnaires before (T0) and after yoga therapy for IG versus waiting period for CG (T1).

**Results:**

A stronger reduction of general fatigue (*P* = .033), physical fatigue (*P* = .048), and depression (*P* < .001) as well as a stronger increase in QoL (*P* = .002) was found for patients who attended 7 or 8 sessions compared with controls. Within the yoga group, both higher attendance rate and lower T0-fatigue were significant predictors of lower T1-fatigue (*P* ≤ .001). Exploratory results revealed that women with breast cancer report a higher reduction of fatigue than women with other types of cancer (*P* = .016) after yoga therapy.

**Conclusion:**

The findings support the assumption that yoga therapy is useful to reduce cancer-related fatigue, especially for the physical aspects of fatigue. Women with breast cancer seem to benefit most, and higher attendance rate results in greater reduction of fatigue.

**Trial registration:**

German Clinical Trials Register DRKS00016034

## Background

The overall survival rate in cancer is rising steadily due to better early detection and treatment options [1]. Therefore, not only the treatment itself but also side effects of cancer and its treatment are gaining more and more attention. Cancer-related fatigue is one of the most common side effects of cancer, which is perceived by patients as even more distressing than pain [2]. Fatigue is characterized by an intense and chronic sense of tiredness that is not associated with previous stress and cannot completely be eliminated by rest. On the physical level, fatigue expresses as tiredness and lack of bodily strength, on the emotional level as demotivation and depressed mood, and on the cognitive level as difficulty concentrating. It considerably reduces the quality of life [3] and prevents patients from living a normal life [4]. Fatigue poses a high risk for the development of clinical depression and anxiety [5] due to the loss of positive experience and increasing avoidance behavior. Prevalence and subjective evaluation of fatigue differ between different types of cancer: patients with breast or colorectal cancer report a higher burden of fatigue than prostate cancer patients [6].

Despite the high prevalence and impairments, the underlying mechanisms are not fully understood. A multimodal etiology including physical and psychosocial factors is assumed. On the physical level, pro-inflammatory cytokines [7–9], hypothalamic-pituitary-adrenal (HPA) axis dysregulation [7], and skeletal muscle dystrophy [10] might play a role. On the psychosocial level, low social and emotional support, low income, and high catastrophizing thoughts can be risk factors for fatigue [11, 12]. As the etiology of fatigue comprises multiple levels, a multimodal approach to reduce fatigue is recommended in various meta-analyses [13]. According to NCCN guidelines, non-pharmacological interventions, such as physical activation management, cognitive behavioral therapy, or mind-body interventions, should be applied before pharmacological ones [14]. Moderate effects of mind-body interventions such as yoga on fatigue are reported in meta-analyses [15, 16]. Yoga combines both psychological (“Mind”) and physical (“Body”) aspects and is becoming increasingly important in supportive cancer research [17]. Even compared with a supportive or psychoeducative group of patients with fatigue, yoga therapy showed significant effects in reducing fatigue symptoms [18]. Besides direct effects on the self-reported fatigue symptoms, yoga also showed positive effects on the assumed physical background by reducing pro-inflammatory cytokines [19–21], decreasing salivary cortisol level, and restoring HPA balance [22–24]. It also helped to increase muscle strength and to reduce musculoskeletal symptoms such as muscle pain [25].

Since breast cancer patients in particular are highly motivated for mind-body interventions, especially yoga, [26] previous studies have examined the effect of yoga therapy on physical and psychosocial aspects predominantly in breast cancer patients with a low sample size [18, 19, 22, 27, 28]. Although other types of cancer suffer just as much from fatigue as breast cancer patients [6], no study has compared the effect of yoga on different types of cancer using the same protocol. As recommended by Lin et al., 2018 [29], we included other types of cancer, which enabled us to compare the efficacy of yoga therapy in the well-evaluated group of women with breast cancer versus women with other types of cancer.

This led to the following research questions:

1. The primary aim of this study was to assess the changes in self-reported fatigue after an 8-week yoga intervention compared with a CG with no intervention for patients with different types of cancer. We expected a higher reduction of fatigue following the yoga intervention compared with CG.
2. For IG, we hypothesized that reduction of fatigue score is higher for patients with a higher attendance rate.
3. In this present study, we examined the difference in efficacy between women with breast (BC) vs. non-breast cancer (NBC) patients.

## Methods

### Trial design

A randomized controlled trial examining the efficacy of yoga compared with a control group for decreasing fatigue symptoms in oncological patients was conducted at radiotherapy outpatient clinic (RAD) and the interdisciplinary oncological therapy outpatient clinic (IOT) of the University Hospital Würzburg. Oncological patients with fatigue scores ≥ 1 on a scale from 0 to 10 [30] were recruited for a non-binding information event. The information events took place every 4 weeks. Participants, who had signed the consent form and completed the first questionnaire set (T0), were randomly assigned to IG or CG. To ensure that enough patients were randomized to the IG to perform the intervention, a block randomization procedure with an allocation ratio of 1:1 was used. Patients attending the same information event formed one block. The randomization lists with computer-generated numbers were created by the Institute of Clinical Epidemiology of the University of Würzburg. All participants of the study received information after the first event on how to deal with fatigue, e.g., management of energy and activities. The IG started with the yoga intervention 1 week after T0; the CG started 10 weeks after T0. The yoga intervention was conducted for 8 weeks plus an additional class in the ninth week to allow the participants to catch up on a missed session. Primary (fatigue) and secondary outcomes (depression and quality of life) were assessed 10 weeks after the start of yoga intervention (T1) via questionnaires.

### Participants

**Fig. 1:**
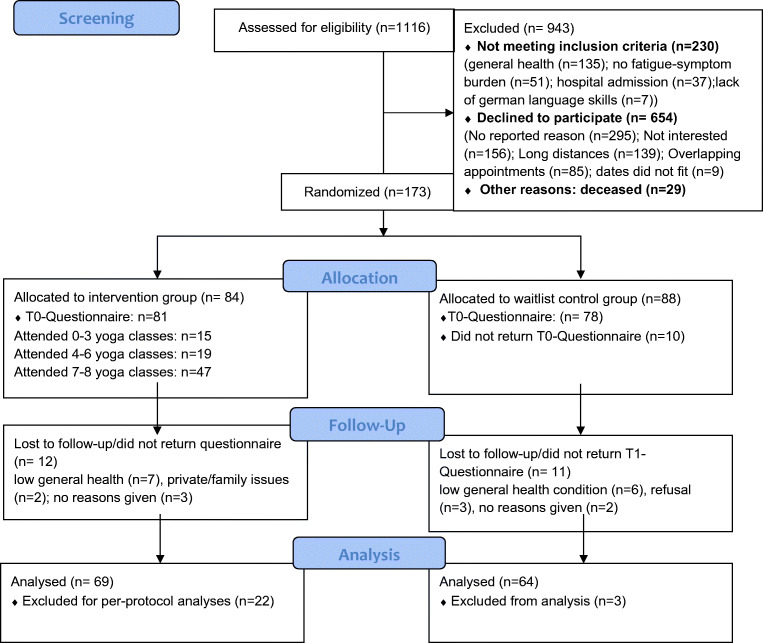
CONSORT diagram showing screening, allocation, and participant flow by group

The participants had to be at least 18 years old, had an oncological disease, were planning to undergo treatment at the time of screening, and had to report at least mild fatigue symptoms (intensity ≥ 1, impairment ≥ 1). Exclusion criteria were insufficient knowledge of German and severe emotional (e.g., severe depressive episode, psychosis) or physical impairment (e.g., unstable metastatic bone disease, acute fractures) as well as more than 50-km distance to the university hospital (due to a high risk of drop-out). Patients were recruited between November 2018 and December 2019. One hundred seventy-three of 1116 eligible patients volunteered to participate (recruitment rate 15.5%), 157 from RAD and 16 from IOT. Reasons—if given—for non-participation were documented (for more information, see Fig. 1).

### Intervention

The yoga therapy consisted of eight 1-h yoga sessions once a week over 8 weeks, which was carried out by certified yoga teachers and was already evaluated in a previous study [31] . Nonviolence (ahimsa) as an essential basic principle of yoga was repeated every class to encourage the participants to be gentle with themselves and accept personal physical limitations. A yoga session consisted of physical postures with awareness (asanas), small series of conscious breathing and deep relaxation (savasana) at the beginning and end of the class (see Table 1). The sequence remained constant for all sessions. In all exercises, participants were reminded to breathe slowly, and consciously. Personal assistance and instructions to adjust the postures and practices were given verbally throughout the class. Participants were encouraged to use tools like rolls or pillows to adapt to individual requirements. The CG received no specific treatment and was asked to complete the questionnaires at the same intervals as IG. After the waiting period, the CG was given the same yoga therapy for 8 weeks.

**Table 1:** Description of yoga class: poses, Sanskrit names and duration of the single poses

	Exercise and description	Sanskrit	Duration (min)
1	Relaxation: conscious breathing, body scan, mindfulness	Savasana	10
2	Vein pump		3
3	Pelvis and back rotation	Nakrasana	3
4	Pelvis opening und strengthening of the abs	Supta Baddha Konasana	4
5	Shoulder bridge strengthening of back and gluteal muscles	Setu Bandhasana	2
6	Seated forward bend pose and variations:	Paschimottanasana and variations with conscious breathing, shoulder stretch	6
7	Backbend: strengthening the muscles in shoulders, arms and hands	Purvottasana	1
8	Diagonal static yoga cat	Majariasana	4
9	Resting pose	Balasana	2
10	Standing exercise/balance exercise		2
11	Upward salute: strengthening of the upper body, training of the balance	Chandrasana	2
12	Warrior 1	Virabhadrasana	4
13	Warrior 3: balance exercise	Virabhadrasana	3
14	Tree: balance Exercise	Vrksasana	4
15	Relaxation	Savasana	10

### Sample size calculation

Two randomized clinical studies investigated similar yoga therapies in cancer patients produced intergroup effect size in fatigue self-assessment scales of *d* = 0.66 [32] and *d* = 0.51 [28]. Based on the smaller effect size of *d* = 0.50, alpha = 0.05, and power = 0.80, a case number of *n* = 64 per group,128 patients in total, was chosen, to conduct a *t* test for independent samples with two-sided testing.

### Measures

Outcomes were assessed using self-report questionnaires at baseline and 10 weeks later after yoga therapy. Sociodemographic data (age, gender, marital status, level of education) and treatment status were assessed at T0. Previous experience with and expectations of yoga were asked at T0. The primary outcome was fatigue. Therefore, German version of EORTC QLQ-FA12 (European Organization for Research and Treatment of Cancer - Quality of Life Questionnaire – Fatigue) [33] was used. This questionnaire can be used for all cancer diseases in all stages and phases of the disease and all areas of treatment (chemotherapy, radiation, surgery) or care (acute care, rehabilitation, aftercare, or palliative care). All items were summed up to one fatigue score and subscores (physical, emotional, and cognitive). Higher scores reflected more fatigue burden. Symptoms of depression, according to DSM-V, were assessed with the Patient Health Questionnaire (PHQ-9) [34], a higher score reflected higher burden of depression. EORTC QLQ-C15-PAL [35] was used to measure quality of life (QoL) for cancer patients and palliative care settings; higher score reflects a higher QoL. For the IG, satisfaction with yoga sessions on different subscales was assessed on a scale 1 (not at all satisfied) to 6 (very satisfied) at the end of the intervention.

### Statistical analysis

For the first research question, repeated measure analyses of variance (ANOVAs) were performed on per-protocol-basis with completers (at least 7 sessions) and intent-to-treat basis using time (T0 vs. T1) as within-factor and type of treatment (IG vs. CG) as between-factor for fatigue and each subscale (physical, emotional, and cognitive), respectively. The second research question was analyzed by linear regression, including attendance rate and T0-fatigue score as independent variable and T1-fatigue score as dependent variable. The third explorative research question was examined by repeated measure ANOVA in women of IG using cancer type (breast vs. non-breast) as between factor.

## Results

### Sample description and baseline data

There were no significant differences between IG and CG in demographic or health-related characteristics. Participants’ age ranges from 24 to 84 (m = 60.4, SD = 11.6), 69.8% were female, and 66% were married or partnered. The participants were predominantly diagnosed with breast cancer (49.1%), followed by prostate cancer (11.9%) and gastrointestinal cancer (10.1%). Seventy-six percent were under cancer-related treatment at T0 (see Table 2). There was no significant difference in T0-fatigue between patients still in treatment and those who had currently no treatment.

**Table 2:** Demographic and clinical characteristics of study population by group. *IG* intervention group, CG control group, SD standard deviation, CNS central nervous system

Characteristics	All (*N* = 159)	IG (*N* = 81)	CG (*N* = 78)
**Age (mean(SD))**	60.4 (11.6)	59.9 (11.7)	60.9 (10.9)
Range	24–84	36–82	24–84
	**% (*N*)**	**% (*N*)**	**% (*N*)**
**Female**	69.8 (111)	67.9 (55)	71.8 (56)
**Marital status**			
Married/partnered	66.0 (105)	71.6 (58)	60.3 (47)
Never Married/single	13.8 (22)	14.8 (12)	12.8 (10)
Divorced/separated	10.7 (17)	7.4 (6)	14.1 (11)
Widowed	6.9 (11)	4.9 (4)	8.9 (7)
**Education level**			
Primary education	29.6 (47)	25.6 (21)	33.3 (26)
Secondary education	30.2 (48)	30.9 (25)	29.5 (23)
Tertiary education	37.1 (59)	40.7 (33)	33.3 (26)
others	3.1 (5)	2.5 (2)	3.8 (3)
**Tumor diagnosis**			
Breast cancer	49.1 (78)	44.4 (36)	53.8 (42)
Prostate cancer	11.9 (19)	16.0 (13)	7.7 (6)
Gastrointestinal cancer	10.1 (16)	12.3 (10)	7.7 (6)
Lung cancer	8.2 (13)	8.6 (7)	7.7 (6)
Lymphoma	8.2 (13)	6.2 (5)	10.3 (8)
Gynecological cancer	4.4 (7)	6.2 (5)	2.6 (2)
Head and neck cancer	3.1 (5)	2.5 (2)	3.8 (3)
Cancer of CNS	2.5 (4)	2.5 (2)	2.6 (2)
Skin cancer	1.3 (2)	1.2 (1)	1.3 (1)
Other cancer	1.2 (2)	0	2.6 (2)
**Therapy during study**	**T0/T1**	**T0/T1**	**T0/T1**
Had a cancer-related therapy	76.1 (121)/ 50.1 (81)	79.0 (64)/ 48.1 (39)	73.1 (57)/ 53.8 (42)
Chemotherapy	15.1 (24)/10.1 (16)	19.8 (16)/11.1 (9)	10.3 (8)/9.0 (7)
Radiation therapy	53.2 (8)3/7.6 (12)	53.1 (43)/6.2 (5)	51.3 (40)/9.0 (7)
Hormone therapy	22.6 (36)/26.5 (42)	22.2 (18)/22.2 (18)	23.1 (18)/30.8 (24)
Antibody therapy	10.1 (16)/12.7 (20)	8.6 (7)/9.9 (8)	11.5 (9)/15.4 (12)
Other	8.2 (13)/ 13.2 (21)	6.2 (5)/ 13.6 (11)	10.3 (8)/ 12.8 (10)
**Treatment intention**	% (*N*)	% (*N*)	% (*N*)
Curative	48.4 (77)	53.1 (43)	43.6 (34)
Palliative	31.4 (50)	28.4 (23)	34.6 (27)
Unknown	20.1 (32)	18.5 (15)	21.8 (17)

### Intervention adherence and evaluation

55.8% of the IG and 50.7% of the CG had no yoga experience at all. Eight participants of the IG and five participants of the CG were practicing yoga before the study. On average, the participants of the IG attended yoga sessions 6.1 (SD = 2.3) times. Fifty-eight percent of participants attended 7–8 sessions. There were no significant differences at baseline between participants who attended 0–6 sessions vs. ≥ 7 sessions (completer) in fatigue, depression, or quality of life.

Participants of the IG were very satisfied with the yoga sessions. Mean ratings on the different subscales (possible range from 1 = “not at all satisfied” to 6 = “very satisfied”) were very high from 4.69 to 5.75. Ninety-five percent would recommend yoga sessions to other patients, and 94.9% would (very) certainly participate in yoga sessions again. No adverse events were reported (see Table 3).

**Table 3:** Adherence and evaluation of yoga sessions. *SD* standard deviation, *NA* not applicable

	Intervention group	Control group
Experience of yoga	%(N)	%(N)
No experience	55.8 (43)	50.7 (35)
Little to moderate experience	39.0 (30)	39.7 (31)
(Very) much experience	5.2 (4)	4.3 (3)
Practicing yoga before the study	9.8 (8)	6.4 (5)
Mean (SD) number of yoga classes attended	6.1 (2.3)	NA
≤ 3 yoga classes	18.5 (15)	NA
4–6 yoga classes	23.5 (19)	NA
≥ 7 yoga classes	58.0 (47)	NA
Evaluation	Mean(SD)	
The selection and combination of exercises?	5.58 (0.66)	NA
The overall structure of a yoga class?	5.75 (0.47)	NA
The length/duration of a therapy session?	5.58 (0.82)	NA
The length of the whole therapy (8 weeks)?	4.71 (1.35)	NA
The instruction by the yoga teachers?	5.86 (0.35)	NA
The group size?	5.56 (0.71)	NA
The possibility to exchange experiences?	4.69 (1.21)	NA
The atmosphere/well-being in the group?	5.48 (0.73)	NA
The breathing exercises/pranayama?	5.38 (0.84)	NA
The meditation part?	5.57 (0.79)	NA
Recommendation of yoga classes to other patients	5.94 (0.30)	NA
Further participation in yoga classes	5.73 (0.55)	NA

### Primary outcome: general, physical, emotional, and cognitive fatigue

**Fig. 2:**
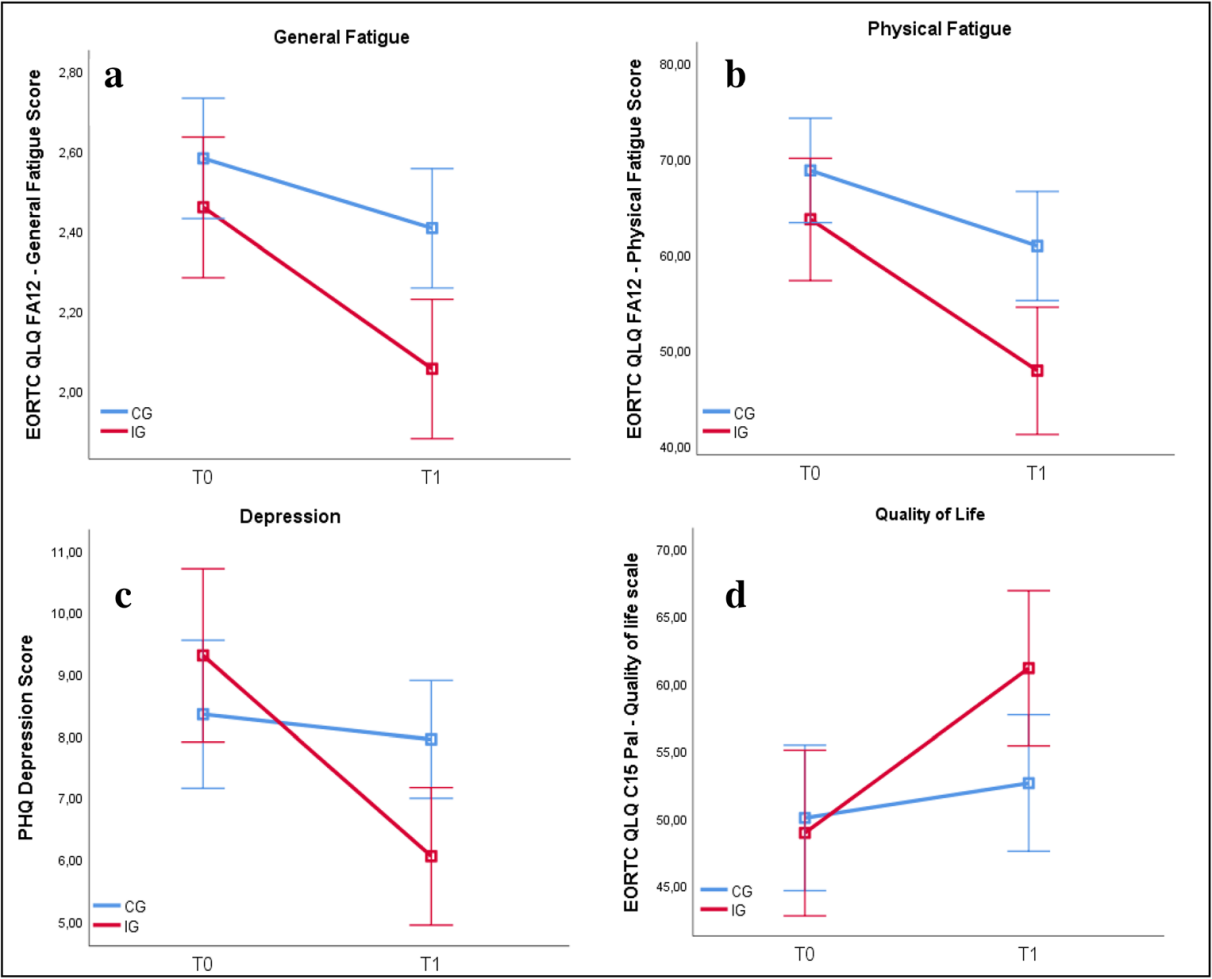
Mean changes in **a** EORTC QLQ FA 12–General Fatigue, **b** EORTC QLQ FA12–Physical Fatigue, **c** PHQ-9 Depression Score, and **d** EORTC QLQ C15 PAL–Quality between T0 und T1 in IG (completers) and CG. Results show mean and 95% CI

Per-protocol analyses with completers compared with controls (IG: *n* = 64; CG: *n* = 47) showed a larger reduction of general fatigue (F(109;1) = 4.66, *P* = .033, *d* = 0.42) as well as physical fatigue (F(109;1) = 4.06, *P* = .048, *d* = 0.39) (see Table 4). In intent-to-treat analyses, less general, physical, and emotional, and cognitive fatigue was reported at T1 compared with T0 (main-effects time (F(131;1) > 5.35, ps < 0.022, *d* > 0.40). On general, physical, and emotional fatigue, IG compared with CG yielded significantly lower scores (F(131;1) > 5.60, ps < 0.02, *d* > 0.41). Although reduction of fatigue did not differ significantly, this effect was explained by attendance rate (for mean scores and significance statistics for fatigue and subscores, see Table 4 and Fig. 2).

**Table 4:** Means (m), standard deviation (SD), and *P* values of ANOVA analyses of time and group effects, and time*group interaction between IG and CG, for per-protocol with completers and intent-to-treat analyses for primary outcome (fatigue and subscales) and secondary outcomes (depression and quality of life)

**Per-protocol analyses**	Yoga group		Control group		Time *P* value	Group *P* value	Time*Group *P* value
	T0 m (SD) *N* = 47	T1 m (SD) *N* = 47	T0 m (SD) *N* = 75	T1 m (SD) *N* = 67			
**Fatigue**	2.5 (0.7)	2.0 (0.5)	2.5 (0.6)	2.4 (0.7)	< .001*	.024*	.033*
**Physical fatigue**	63.6 (25.5)	47.8 (21.9)	67.5 (19.7)	60.1 (24.0)	< .001*	.048*	.020*
**Emotional fatigue**	35.6 (28.4)	27.1 (25.1)	42.1 (24.9)	38.1 (27.1)	.010*	.030*	.530
**Cognitive fatigue**	30.9 (23.9)	18.8 (16.1)	31.8 (22.9)	28.6 (24.6)	< .001*	.089	.110
**Depression**	9.3 (5.2)	6.0 (3.5)	8.0 (4.6)	7.9 (4.1)	< .001*	.540	<.001*
**Quality of life**	48.9 (20.3)	60.9 (18.2)	51.6 (20.9)	53.0 (19.7)	.001*	.275	.026*
**ITT analyses**	Yoga group		Control group		Time *P* value	Group *P* value	Time*group *P* value
	T0 m (SD) *N* = 81	T1 m (SD) *N* = 69	T0 m (SD) *N* = 75	T1 m (SD) *N* = 67			
**Fatigue**	2.5 (0.7)	2.1 (0.6)	2.5 (0.6)	2.4 (0.7)	.001*	.013*	.166
**Physical fatigue**	65.1 (24.8)	49.6 (23.2)	67.5 (19.7)	60.1 (24.0)	.001*	.020*	.124
**Emotional fatigue**	36.6 (29.4)	28.3 (26.0)	42.1 (24.9)	38.1 (27.1)	.022*	.016*	.877
**Cognitive fatigue**	29.8 (23.4)	21.0 (19.1)	31.8 (22.9)	28.6 (24.6)	.001*	.086	.341
**Depression**	8.8 (5.0)	6.1 (3.9)	8.0 (4.6)	7.9 (4.1)	<.001*	.271	.001*
**Quality of life**	50.6 (21.3)	59.5 (19.9)	51.6 (20.9)	53.0 (19.7)	.002*	.081	.256

#### Attendance

Within IG, multiple linear regression with T1-fatigue as dependent variable and T0-fatigue and attendance rate as independent variable had a significant regression equation (F(66;2) = 18.3 *P* < .001) with *R*^2^ = .36. T1-fatigue decreased .11 for each attended class. Both T0-fatigue (*r* = .491) and attendance rate (*r* = − .273) were significant predictors of T1-fatigue (*P* ≤ .001).

Within IG, ANOVA with attendance rate as categorical dependent variable (0–3 vs. 4–6 vs. 7–8 attended sessions) showed a significant time*attendance rate interaction (F(66; 2) = 6.513, *P* = .003). Participants who attended three or fewer sessions had higher general fatigue scores at T1 compared with participants who attended 4–6 sessions and 7–8 sessions.

#### Cancer diagnosis

Within females of IG, there was no significant group effect (*P* = .813) when comparing BC (*N* = 26) vs. NBC (*N* = 20), but significant time effect (F(44;1) = 21.0; *P* < .001) and time*group interaction (F(44;1) = 7.0; *P* = .011). BC patients reported a higher reduction in fatigue between T0 and T1 than NBC patients did.

### Secondary outcomes: depression and QoL

#### Depression

In per-protocol analysis, a significant time*group interaction with high effect size was found (F(109;1) = 16.83; *P* < .001; *d* = .79). Participants in the IG had significant lower depression scores after participating in yoga sessions compared with the CG with medium effect size. Intent-to-treat analysis also revealed a significant time*group interaction (F(131;1) = 10.71; *P* = .001; *d* = .57).

#### Quality of life

In per-protocol analysis, significant group*time interaction with medium effect size was found (F(101;1) = 5.08; *P* = .026; *d* = .45). The IG had a higher QoL after 8 weeks than the CG after this time. Intent-to-treat analysis revealed a significant time effect (F (122;1) = 9.65; *P* = .002; *d* = .56). Participants in the IG had higher QoL with medium size effect (see more in Table 4 and Fig. 2).

## Discussion

This randomized controlled trial examined the efficacy of an 8-week yoga intervention on fatigue in a group of oncological patients with different cancer types. Only few studies have investigated the efficacy of yoga with such a large sample size [36, 37]. In addition to patients diagnosed with breast cancer, this study also included other types of cancers [26, 38].

In per-protocol analyses with completers, significant time*group interactions with a small effect size (0.39–0.42) were found in general fatigue and physical fatigue. However, these effects crucially depend on attendance, intent-to-treat analyses revealed a general decrease, but no significant interaction has been found. More importantly, higher frequency of attended sessions was associated with a more pronounced decrease of fatigue, which supports the findings in previous studies [21]. Significant difference in efficacy has been found between patients who attended 0–3 sessions vs. more (4–6 or 7–8). So at least four participations seem to be useful for decreasing fatigue.

Overall, the results of the study are predominantly in line with the results of more recent meta-analyses, which could only find small to medium effect sizes through yoga therapy regarding fatigue [29]. At this point, it is important to note that a yoga therapy with only 8 1-h sessions is a rather short and small intervention. It can be supposed that a yoga therapy with higher intensity in length, regularity, and frequency of practice would result in bigger effects [21, 28, 32, 39, 40].

In addition, the differences in efficacy concerning different types of cancer must be considered. To the best of our knowledge, there is currently no study comparing different types of cancer in a yoga therapy [18, 19, 22, 27, 28]. In this study, women with BC compared with women with other cancer showed a stronger reduction of fatigue. Patients with BC experience a higher reduction of fatigue through yoga, and thus benefit the BC patients, however report descriptive higher levels of fatigue at all subscales. Therefore, the lower efficacy in NBC women might also be a possible bottom effect, as they already suffer little or less from fatigue. It must also be taken into account that BC patients, as middle-aged women, belong to the group that is very much addressed by yoga [41]. Therefore, it might be possible that expectation effects might contribute to higher benefits in breast cancer patients. In this study, there was no significant difference in self-reported expectation regarding yoga therapy between BC and NBC women (*P* = .460). Nevertheless, this can only be seen explorative since the group of NBC patients is very diverse.

This study differs from other studies by the diversity of the sample. In this study, patients with other types of cancer were included in addition to BC patients, including mainly prostate and gastrointestinal cancer, which also reflects the general cancer prevalence in the general population [42]. Furthermore, this study may benefit from a higher relative number of recruited men of 30% compared with other studies of about 5% [43, 44], which contributes to a better generalizability of the results. Nevertheless, a balanced gender ratio could not be achieved and a selection bias has to be assumed. Women are more interested in complementary alternative medicine, such as yoga or acupuncture than men [45, 46], which can lead to a higher participation and attendance rate and thus to a better efficacy [21]. Nevertheless, the low consent rate of 15.5% must be taken into account for very broad inclusion criteria (fatigue ≥ 1). However, the inclusion criterion of fatigue was deliberately chosen to be so broad, as 90% of cancer patients undergoing therapy suffer from fatigue during the course of treatment [19]. Therefore, it seems to be appropriate to offer yoga therapy at the earliest possible point in treatment, if there is no contradiction against yoga for each single patient, so that patients can learn helpful techniques for the prophylactic management of fatigue.

The diversity of the sample must also be viewed critically. Cancer subgroups cannot be validly compared with each other due to the very different sample sizes. Further research with same sample sizes in the different types of cancer is certainly necessary. The treatment status has also to be considered. Some of the participants were still under treatment at the beginning of the study (*N* = 121); others had already completed treatment (*N* = 34). A worsening of the fatigue symptoms during the study can also be due to the presence of treatment or an improvement in the symptoms due to the absence of treatment [47]. Studies involving both patients during and after treatment had less consistent and significant findings regarding fatigue [39, 47]. In this sample, there was no significant association between treatment status and baseline fatigue. Thus, in this study, the influence of treatment status on T0 in terms of fatigue can be considered negligible. Furthermore, due to the lack of an active waiting control group, it cannot be excluded that the effects found are due to unspecific factors, such as the influence of the group or the mere activation by a regular weekly appointment. Thus, only an efficacy of the yoga therapy can be investigated, but not an efficiency compared with other interventions [48].

The study shows that yoga therapy is feasible and accepted in and very well evaluated by patients with breast and other types of cancer. It has positive effects on physical and general fatigue if the attendance rate is sufficient. The influence of the type of cancer on the effectiveness of the yoga therapy cannot be clarified due to the very small subgroups. In addition, studies with an active control group such as walking or psychoeducation would be helpful to shed light on the effectiveness of yoga.

Yoga therapy should continue to be offered, evaluated, and expanded as supportive therapy. Further investigation is needed to explore factors, which might possibly hinder or help in practicing yoga. As attendance rate plays an important role for the effectiveness of yoga, new ways as online or video-supported yoga sessions, combined with regular reminders, could be promising and should be explored in future studies, as they could help to overcome barriers to a regular self-practice.

## Data Availability

Not applicable

## Abbreviations

BC: breast cancer
CG: control group
IG: intervention group
IOT: interdisciplinary oncological therapy outpatient clinic
NBC: non-breast cancer
PHQ-9: Patient Health Questionnaire
QoL: quality of life
RAD: radiotherapy outpatient clinic
